# Identification of biomarkers associated with synovitis in rheumatoid arthritis by bioinformatics analyses

**DOI:** 10.1042/BSR20201713

**Published:** 2020-09-18

**Authors:** Zhaoyan Li, Meng Xu, Ronghang Li, Zhengqing Zhu, Yuzhe Liu, Zhenwu Du, Guizhen Zhang, Yang Song

**Affiliations:** 1 Department of Orthopedics of the Second Hospital of Jilin University, Ziqiang Street 218, Changchun, Jilin 130041, China; 2 Research Centre of the Second Hospital of Jilin University, Ziqiang Street 218, Changchun, Jilin 130041, China; 3 The Engineering Research Centre of Molecular Diagnosis and Cell Treatment for Metabolic Bone Diseases of Jilin Province, Ziqiang Street 218, Changchun, Jilin 130041, China

**Keywords:** bioinformatics, hub gene, Rheumatoid arthritis

## Abstract

**Objectives:** Rheumatoid arthritis (RA) is the most common inflammatory arthritis in the world, but its underlying mechanism is still unclear. The present study aims to screen and verify the potential biomarkers of RA.

**Methods:** We searched the Gene Expression Omnibus (GEO) database for synovial expression profiling from different RA microarray studies to perform a systematic analysis. Functional annotation of differentially expressed genes (DEGs) was conducted, including GO enrichment analysis and Kyoto Encyclopedia of Genes and Genomes (KEGG) pathway enrichment analysis. The protein–protein interaction (PPI) networks of the DEGs were constructed based on data from the STRING database. The expression levels of the hub genes in normal membranes and RA synovium were detected by quantitative real-time polymerase chain reaction (qRT-PCR) and Western blot system.

**Results:** A total of 444 differential expression genes were identified, including 172 up-regulated and 272 down-regulated genes in RA synovium compared with normal controls. The top ten hub genes; protein tyrosine phosphatase receptor type C (*PTPRC*), LCK proto-oncogene (*LCK*), cell division cycle 20 (*CDC20*), Jun proto-oncogene (*JUN*), cyclin-dependent kinase 1 (*CDK1*), kinesin family member 11 (*KIF11*), epidermal growth factor receptor (epidermal growth factor receptor (*EGFR*), vascular endothelial growth factor A (*VEGFA*), mitotic arrest deficient 2 like 1 (*MAD2L1*), and signal transducer and activator of transcription 1 (*STAT1*) were identified from the PPI network, and the expression level of VEGFA and EGFR was significantly increased in RA membranes (*P*<0.05).

**Conclusion:** Our results indicate that the hub genes *VEGFA* and *EGFR* may have essential effects during the development of RA and can be used as potential biomarkers of RA.

## Introduction

Rheumatoid arthritis (RA) is a chronic, aggressive, and progressive autoimmune disease, which can cause bone and cartilage damage as well as disability [1]. The main pathological features of RA are joint involvement, proliferative synovitis, destruction of bone and cartilage. The occurrence and development of RA involve many factors, which are related to various factors, such as genetic factors, infection, and immune dysfunction [2]. Although there is extensive research into the mechanisms and causes of RA formation and progression, the etiology of RA is still not clear [3].

Recent research has found that changes in genetic factors contribute to the development of rheumatic diseases and are supported by twin studies [4]. RA is a highly heterogeneous disease whose results are difficult to predict. The degree of joint damage in RA patients varies widely from individual to individual. Many patients can achieve remission if they are detected early and continue to be treated promptly. Patients with RA must be treated with disease-modifying anti-rheumatic drugs (DMARDs) that improve symptoms. The combination of biologic DMARDs and targeted synthetic DMARDs has higher efficacy than single-use prescriptions [5,6]. Understanding the intrinsic relationship among immunity, genetic and environmental factors are the key to explaining the cause of RA [7]. The genome-wide association study (GWAS) has found many susceptibility genes for RA in European, Asian, and other ethnic groups [8]. At the same time, many studies have been done on RA with the development of high-throughput sequencing technology, and thousands of differentially expressed genes (DEGs) have been screened. However, due to different sequencing platforms or sample heterogeneity, the results of gene expression profiles are inconsistent with different gene profiles.

In the present study, we combined bioinformatics analysis and molecular biology identification to identify the hub genes involved in RA. We have downloaded three microarray datasets GSE55457, GSE55235, and GSE12021 from the Gene Expression Omnibus (GEO) database. A total of 35 RA samples and 29 normal controls were included in the study. DEGs between RA synovium and normal controls were screened out by the bioinformatics method. Gene ontology (GO) and pathways enrichment analyses of DEGs were applied, and the protein–protein interaction (PPI) network of these DEGs was constructed by Cytoscape software. The purpose of the present study was to identify hub genes and pathways of RA and then to explore the potential biomarkers for early diagnosis and treatment of RA.

## Materials and methods

### Gene expression profile data

The GEO dataset is a public repository database, which stores a large number of gene functions and expression datasets [9]. The raw data of GSE55457, GSE55235, and GSE12021 (GPL96 platform) were downloaded from the GEO database. The microarray data of GSE55457 include 13 RA synovium and 10 normal knee synovium, the microarray data of GSE55235 include 10 RA synovium and 10 normal knee synovium [10], and the microarray data of GSE12021 include 12 RA synovium and 9 normal knee synovium [11].

### Microarray data analysis

The DEGs of the RA synovium and the normal synovium was identified by the limma package in R [12]. The data pre-processing process includes background adjustments, normalization, and summarization. The integration of DEGs were analyzed by the FunRich software [13].

### GO and pathway enrichment analysis

GO is an annotation method based on high-throughput genomic or transcriptome data to enrich genes. GO analysis includes categories of biological processes (BPs), molecular function (MF), and cellular component (CC). Pathway analysis is a functional analysis that maps genes to Kyoto Encyclopedia of Genes and Genomes (KEGG) pathways. GO and KEGG analyses were performed using the online DAVID (The Database for Annotation, Visualization and Integrated Discovery) bioinformatics database (https://david.ncifcrf.gov/) [14]. Gene count > 2 and *P*<0.05 were set as the cut-off point.

### PPI network construction

Proteins and their functional interactions are the backbones of cellular mechanisms. STRING is an online protein database which can be used to collect and integrate ‘protein–protein’ interaction resources [15]. The interaction scores > 0.4 were defined as significant. Afterward, the raw data were imported into Cytoscape software to build a PPI network.

### Validation of gene expression

The top ten hub genes were verified by the quantitative real-time polymerase chain reaction (qRT-PCR). Total RNA was extracted from the control and RA samples using TRIzol reagent (Invitrogen, Carlsbad, U.S.A.) and then reverse transcribed to cDNA. The expression levels of mRNAs were further assessed by qRT-PCR using QSYBR Green Supermix (Bio-Rad, Hercules, CA, U.S.A.) with a QuantStudio™ 7 Flex RT-PCR system (Applied Biosystems, Carlsbad, U.S.A.). Primers were designed by Primer Premier 5.0 Software (PREMIER Biosoft, Palo Alto, U.S.A.) that are shown in Table 1. All samples were normalized to the expression of glyceraldehyde-3-phosphate dehydrogenase (GAPDH), and the experiment was repeated three times. Relative expression levels are calculated using the 2^−ΔΔ*C*_T_^ method, and statistical analysis was performed using SPSS software (version 22.0).

**Table 1:** The primers of top ten hub genes

Gene	Forward primer	Reverse primer
*GAPDH*	CGGACCAATACGACCAAATCCG	AGCCACATCGCTCAGACACC
*PTPRC*	AACAGTGGAGAAAGGACGCA	GGCAAAGCCAAATGCCAAGA
*LCK*	CCAGGATCTCACAATCTCAGGG	ATAACCAGGTTGTCTTGCAGT
*CDC20*	GCAAGGAGAACCAGCCTGAA	TGATAACCCTCTGGCGCATT
*JUN*	GAGCTGGAGCGCCTGATAAT	CCCTCCTGCTCATCTGTCAC
*CDK1*	TTTCTTTCGCGCTCTAGCCA	GGTAGATCCGCGCTAAAGGG
*KIF11*	TCCCCGTAACAAGAGAGGAGT	TCCTTTTTGCTGCCCCCTTT
*EGFR*	AAATGGGCTGCAAAGCTGTC	TTCCAGACAAGCCACTCACC
*VEGFA*	GTGAATGCAGACCAAAGAAAGA	AGGCTCCAGGGCATTAGAC
*MAD2L1*	CGTGCTGCGTCGTTACTTTT	GCCGAATGAGAAGAACTCGG
*STAT1*	ACCTAACGTGCTGTGCGTAG	TGAGACATCCTGCCACCTTG

### Western blot assay

The expression of vascular endothelial growth factor A (VEGFA) and epidermal growth factor receptor (EGFR) were detected by Western blotting. RA synovitis was lysed in IP cell lysis buffer on ice. The quantitative protein was denatured and transferred to the polyvinylidene difluoride membranes. The membranes were blocked with 5% bovine serum albumin (BSA) for 2 h at room temperature, the polyvinylidene fluoride (PVDF) membrane was incubated with the BSA first antibody overnight at 4°C. After washing the membrane with Tris-buffered saline Tween (TBST), it was incubated with the secondary antibody for 2 h at room temperature. The β-actin was set as an internal control. The primary antibody of VEGFA (1:1000), EGFR (1:1000), and β-Actin (1:1000) used in the present study were purchased from Signalway Antibody LLC (Maryland, U.S.A.). Protein bands were visualized by Azure Biosystems C600 (Azure Biosystems, U.S.A.).

### Case and control groups

Our study was approved by the Ethics Committee of the Second Hospital of Jilin University, Jilin, China. A total of ten patients with RA were included in the present study, including four males and six females aged 49.9 ± 4.21. There were ten donors in the control group, including four males and six females, aged 47.8 ± 2.99. Informed consent was obtained from all enrolled groups. RA synovial tissue was obtained from patients undergoing total knee arthroplasty at The Second Hospital of Jilin University, and normal synovial tissue was taken from donors undergoing knee arthroscopy at The Second Hospital of Jilin University.

### Statistical analysis

Each experiment was repeated at least three times, and the data were presented as the mean ± standard deviation. Comparisons between groups were performed using unpaired Student’s *t* test. *P*-values <0.05 were considered statistically significant.

## Results

### Identification of DEGs in RA

**Figure 1:**
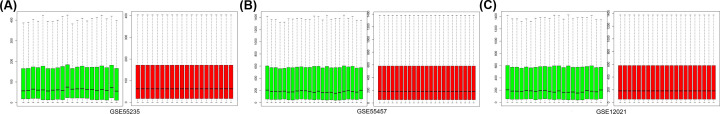
Boxplots of sample data prior to and following normalization The green box plots represent the data before normalization, and the red box plots represent the normalized data. (**A**) Boxplots of GSE12021. (**B**) Boxplots of GSE55235. (**C**) Boxplots of GSE55457.

**Figure 2:**
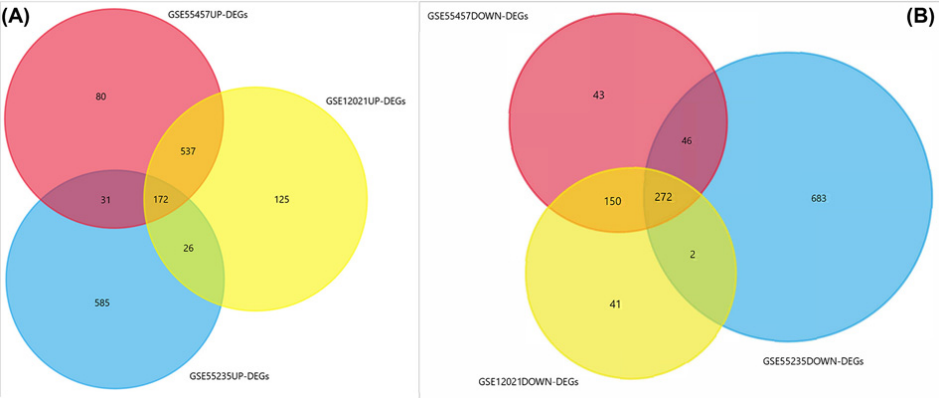
Venn diagram of three cohort profile datasets, and different color areas represented different datasets (**A**) Up-DEGs of three cohort profile datasets. (**B**) Down-DEGs of three cohort profile datasets.

A total of 35 RA samples and 29 normal controls were included in the study. The raw data of GSE55457, GSE55235, and GSE12021 dataset were standardized, and the results are shown in Figure 1. The |log2(fold change)| > 1 and *P*-value <0.05 were used to evaluate significant differences in the expressions of mRNA between the two groups, we extracted 1331, 1817, and 1325 differential expression genes from the expression profile datasets GSE55457, GSE55235, and GSE12021, respectively. Integration of DEGs, a total of 444 differential expression genes were identified, including 172 up-regulated and 272 down-regulated genes in RA synovium compared with normal controls (Figure 2).

### Enrichment analysis of DEGs

**Figure 3:**
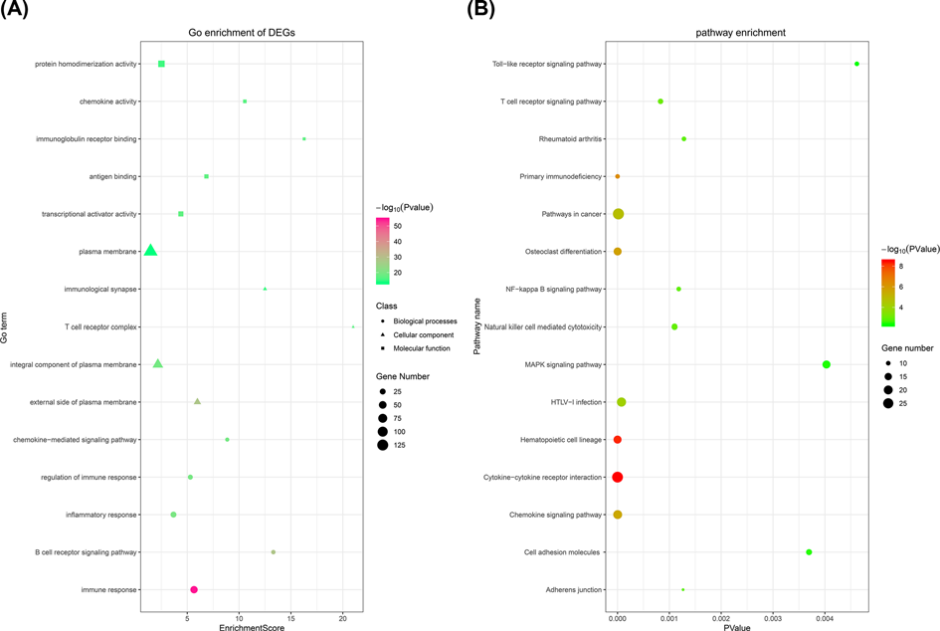
Network and pathway analyses of DEGs (**A**) The top five GO terms in each group: the gradual color represents the *P*-value; the dot represents BPs, the triangle represents CC, the square represents MF, and the size of the black spots represents the gene number. (**B**) KEGG enrichment analysis of the DEGs. The gradual color represents the *P*-value, and the size of the black spots represents the gene number. GO and KEGG enrichment analyses were analyzed by the DAVID database.

**Figure 4:**
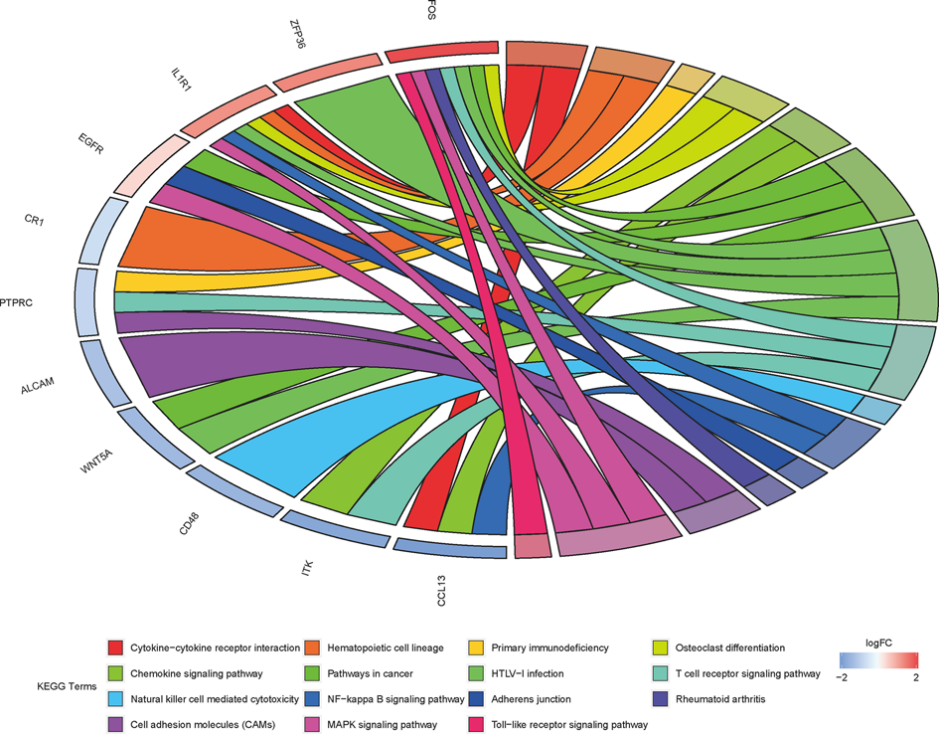
KEGG enrichment analysis of DEGs: the gradual color represents the log FC

To acquire the gene functions of DEGs, gene function enrichment analysis was analyzed by the DAVID database. These DEGs were classified into three groups as follows: BP, CC, and MF. The top five BP, CC, and MF terms are shown in Table 2 and Figure 3A. In the BPs group, the DEGs are mainly enriched in the immune response, B-cell receptor signaling pathway, inflammatory response, regulation of immune response, and chemokine-mediated signaling pathway. In the CC group, the DEGs are mainly enriched in the T-cell receptor complex, immunological synapse, external side of plasma membrane, integral component of plasma membrane, and plasma membrane. Moreover, in the MF group, the DEGs are mainly enriched in chemokine activity, transcriptional activator activity, antigen binding, immunoglobulin receptor binding, and protein homodimerization activity. KEGG analysis shows that DEGs were enriched in pathways such as Cytokine–cytokine receptor interaction Osteoclast differentiation, Chemokine signaling pathway, NF-κB signaling pathway, RA, MAPK signaling pathway, and Cell adhesion molecules. The results were shown in Figures 3B and 4, and Table 3.

**Table 2:** The significant enriched analysis of DEGs in RA

Expression	Category	Term	Description	Gene count	*P*-value
DEGs	BP	GO:0006955	Immune response	53	2.68E-24
	BP	GO:0050853	B-cell receptor signaling pathway	16	4.71E-13
	BP	GO:0006954	Inflammatory response	31	1.82E-09
	BP	GO:0050776	Regulation of immune response	21	2.90E-09
	BP	GO:0070098	Chemokine-mediated signaling pathway	14	4.72E-09
	CC	GO:0009897	External side of plasma membrane	27	5.73E-13
	CC	GO:0005887	Integral component of plasma membrane	65	4.87E-09
	CC	GO:0042101	T-cell receptor complex	8	4.63E-08
	CC	GO:0001772	Immunological synapse	9	4.14E-07
	CC	GO:0005886	Plasma membrane	128	1.59E-06
	MF	GO:0001077	Transcriptional activator activity	22	3.35E-08
	MF	GO:0003823	Antigen binding	15	3.61E-08
	MF	GO:0034987	Immunoglobulin receptor binding	9	4.24E-08
	MF	GO:0008009	Chemokine activity	11	6.39E-08
	MF	GO:0042803	Protein homodimerization activity	39	3.20E-07

GO enrichment analysis was analyzed by the DAVID database.

**Table 3:** Signaling pathway enrichment analysis of DEGs function in RA

Expression	Term	Description	Gene count	P-value
DEGs	hsa04060	Cytokine–cytokine receptor interaction	28	3.19998E-09
	hsa04640	Hematopoietic cell lineage	17	4.88504E-09
	hsa05340	Primary immunodeficiency	10	4.83473E-07
	hsa04380	Osteoclast differentiation	17	1.75917E-06
	hsa04062	Chemokine signaling pathway	20	2.8964E-06
	hsa05200	Pathways in cancer	29	1.71096E-05
	hsa05166	HTLV-I infection	21	7.59384E-05
	hsa04660	T-cell receptor signaling pathway	11	0.000829754
	hsa04650	Natural killer cell-mediated cytotoxicity	12	0.001100152
	hsa04064	NF-κB signaling pathway	10	0.001180749
	hsa04520	Adherens junction	9	0.001264439
	hsa05323	RA	10	0.001281492
	hsa04514	Cell adhesion molecules	12	0.003694171
	hsa04010	MAPK signaling pathway	17	0.004030366
	hsa04620	Toll-like receptor signaling pathway	10	0.004617177

Abbreviation: CAM, cell adhesion molecule. KEGG enrichment analysis was analyzed by the DAVID database.

### PPI network analysis and hub genes screening

**Figure 5:**
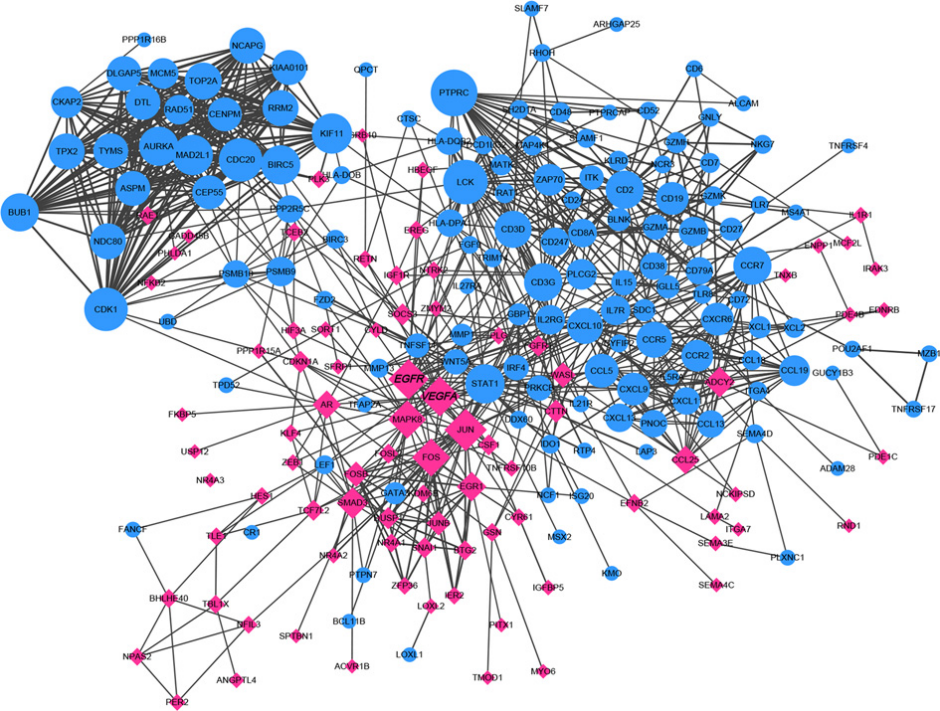
The PPI network of DEGs: red squares represent up-regulated DEGs, and blue circles represent down-regulated DEGs The size of the squares and circles represents the degree value. PPI was analyzed by the STRING database.

Based on the STRING online database, we constructed a PPI network with Cytoscape software, containing 222 nodes and 933 edges (Figure 5). We used the degree analysis method of plug-in CytoHubba in Cytoscape to screen the hub genes. The results show that the top ten genes, which were mostly connected with other genes or proteins, including protein tyrosine phosphatase receptor type C (*PTPRC*), LCK proto-oncogene (*LCK*), cell division cycle 20 (*CDC20*), Jun proto-oncogene (*JUN*), cyclin-dependent kinase 1 (*CDK1*), kinesin family member 11 (*KIF11*), *EGFR, VEGFA*, mitotic arrest deficient 2 like 1 (*MAD2L1*), and signal transducer and activator of transcription 1 (*STAT1*), were identified as hub genes.

### Validation of hub genes expression level

**Figure 6:**
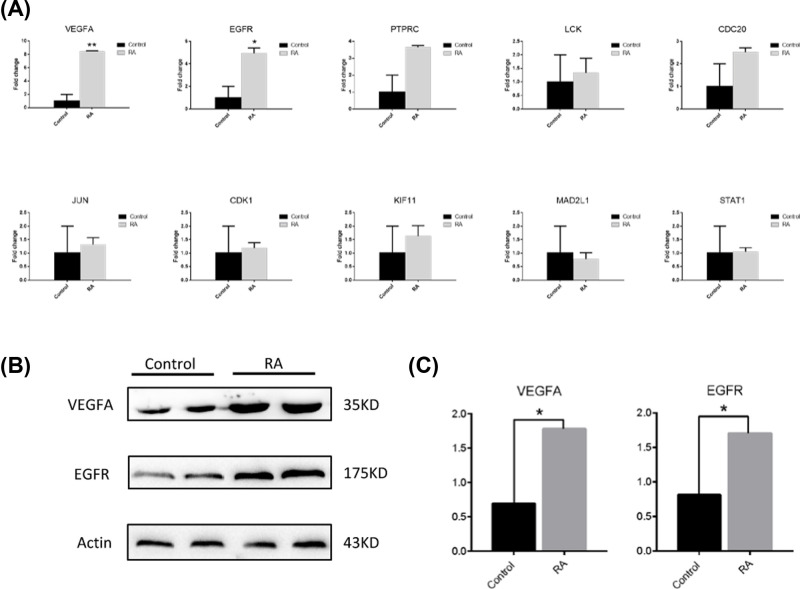
The expression level of hub genes in RA synovial tissue and control group (**A**)Validation of the top ten hub genes by qRT-PCR between the RA group and the control group. * represents *P*<0.05, and ** represents *P*<0.01. (**B**) The expression level of VEGFA and EGFR in the RA group and control group. (A) Western blotting detection of VEGFA and EGFR in RA synovial membrane and normal synovial membrane. (**C**) The expression level of Western blotting detection. * represents *P*<0.05.

To verify the results of bioinformatics analysis, the qRT-PCR system was used to detect the expression levels of the top ten hub genes in RA synovial tissue and control groups. Statistical results indicate that the expression levels of EGFR and VEGFA in RA synovial tissue were significant (*P*<0.05) (Figure 6A). Moreover, to further verify whether these genes were highly expressed in RA, the expression of EGFR and VEGFA were examined by Western blot in RA synovial tissue and normal synovial tissues, and the results showed that the expression levels of VEGFA and EGFR in the synovial tissue of RA were significantly increased (*P*<0.05). The results were shown in Figure 6B,C.

## Discussion

RA is the most common inflammatory arthritis in the world, affecting approximately 1% of the world’s population [16]. The incidence of RA increases with age, however, most commonly between 50 and 60 years, usually around 30 years [17]. Early treatment of RA has been shown to prevent irreversible dysfunction and tissue damage, so early identification and treatment of RA are critical for best outcomes [18]. Therapeutic antibodies against inflammatory cytokines or immune cells have become an essential strategy for RA treatment. However, the current molecular biology research of RA cannot provide sufficient data for biotherapy [19]. In the past two decades, the biotherapy of RA has made significant progress. Information obtained from human genomics and proteomics projects has led to a better understanding of the etiology and pathogenesis of the disease and has made significant advances in its treatment.

The genomics studies in RA mostly focus on a single genetic event or single cohort study. Our study integrated three profile datasets from GEO database and used bioinformatics tools to analyze microarray data. A total of 444 DEGs were detected, including 172 up-DEGs and 272 down-DEGs, and we performed GO function analysis and pathway analysis of the DEGs, respectively. Next, we constructed a PPI network for DEGs using the STRING database, and the top ten hub genes that were further analyzed by Cytoscape software as follows: *PTPRC, LCK, CDC20, JUN, CDK1, KIF11, EGFR, VEGFA, MAD2L1*, and *STAT1*. The statistical results validated by the qRT-PCR and Western blot system show that the expression levels of VEGFA and EGFR were significantly increased in RA samples. As potential biomarkers, VEGFA and EGFR are very important for early diagnosis and clinical treatment of RA, because they can help predict the disease development of subjects at risk, provide prognostic information that can be used to make treatment choices and evaluate treatment response and results, and can monitor disease activity and progress.

Angiogenesis is a prominent feature of RA. The formation of new blood vessels provides oxygen and nutrients to the inflammatory cells, which contributes to the persistence of joint diseases [20]. VEGFA is a critical factor in vascular development, and it is a dominant endothelial growth factor, which is up-regulated by proinflammatory cytokines and hypoxia. It has been reported that VEGFA inhibitors block the new blood vessels of RA and inhibit the transmission of nutrient proteins to the site of inflammation [21]. In our study, VEGFA has significantly increased in RA samples. The KEGG analysis showed that VEGFA was enriched in the RA pathway. The level of VEGFA in the joint fluid of patients with RA was significantly higher than the normal control group. Serum VEGFA level was closely related to RA disease activity, especially related to joint swelling [22]. Previous studies showed that the VEGFA rs699947 C/A functional polymorphism might be associated with the risk of RA in elderly patients [23]. The genetic variation of the VEGFA gene is associated with circulating VEGF-A levels in RA patients [24]. In our previous research, the expression levels of VEGFA have significantly increased in knee osteoarthritis synovial membranes [25,26]. Angiogenesis is essential for the expansion of synovial tissue in RA: pre-existing blood vessels promote blood-derived leukocytes to enter the subsynovial layer, producing and enhancing inflammation. Angiogenesis involves multiple steps, and each step is regulated by specific factors [27]. The inflammation in osteoarthritis is different from the inflammation in RA. The pathogenesis of osteoarthritis is characterized by chronic low-grade inflammation in the synovium, whereas patients with RA manifest with persistent, high-grade systemic inflammation [28,29]. Previous studies have found that the expression of VEGF in all layers of the synovial tissue of RA is much higher than that of osteoarthritis, which may be due to the high expression of VEGF in early RA synovial tissue, which promotes the formation of neovascularization. At the same time, a large number of other inflammatory factors are expressed around the blood vessels, which directly leads to the tortuous proliferation of blood vessels [30]. Many anti-rheumatic drugs used clinically affect the vascular structure; cyclosporine A has recently been shown to inhibit endothelial cell migration and angiogenesis induced by VEGF, which may be a new mechanism of cyclosporine A in the treatment of RA [20,31]. Targeting newly formed RA vasculature may alter disease progression, and VEGFA inhibitors that inhibit neovascularization may be a new target for RA therapy. The levels of IL-6 and VEGFA in the serum of patients with RA vary with the progression of the disease, which may be a rheumatoid effective disease marker [32].

EGFR is a receptor tyrosine kinase that plays a vital role in developing various tumors [33]. The pathological process of RA is similar to tumors, involving both neovascularization and inflammatory cell infiltration. EGFR acts as a mediator of synovial hyperplasia, which includes many aspects of angiogenesis and regulation [34]. Therefore, EGFR is also involved in the progression of RA. EGFR plays an essential role in the pathogenesis of RA by promoting angiogenesis, cytokine production of synovial fibroblasts, and proliferation of endothelial cells [35]. The treatment of RA mainly inhibits the immune system and inhibits synovial and vascular proliferation by blocking the EGFR signaling pathway, which can provide synergy with current therapies. In our study, EGFR has significantly increased in RA samples, and the KEGG analysis showed that EGFR was mainly enriched in adherens junction, MAPK signaling pathway, and ErbB signaling pathway. EGFR is highly expressed in the synovial tissue of RA patients and autoimmune arthritis mice. Also, the study found that the EGFR inhibitor erlotinib can reduce autoimmune arthritis in mice, reducing synovial membrane and vascular membrane formation [36]. Previous studies have found a statistically significant difference in the genotype frequency distribution between the RA patients and the control group at the EGFR rs17337023 SNP locus. Moreover, EGFR gene rs17337023 polymorphism is associated with the risk of RA in the Iranian population [37,38]. In conclusion, EGFR and its ligands may be involved in the development of RA. The development of therapeutic drugs targeting EGFR will bring a new treatment plan for the RA [39].

Taken above, using bioinformatics tools and tissue sample verification, we have identified two candidate genes and pathways, and these findings may significantly improve our understanding of the causes and potential molecular events of RA, and the hub genes VEGFA and EGFR maybe have a crucial function in the pathogenesis of RA and could be used as potential biological and therapeutic indicators of RA. There are still many limitations in our study: small tissue sample size was used for the analyses and mRNA to protein synthesis is regulated by many factors and lack of *in vivo* experiment. To further verify the experimental results, more experimental *in vivo* with extensive well-designed studies are needed.

## Abbreviations

BP: biological process
BSA: bovine serum albumin
CC: cellular component
CDC20: cell division cycle 20
CDK1: cyclin-dependent kinase 1
DAVID: The Database for Annotation, Visualization and Integrated Discovery
DEG: differentially expressed gene
DMARD: disease-modifying anti-rheumatic drug
EGFR: epidermal growth factor receptor
GEO: Gene Expression Omnibus
GO: Gene Ontology
JUN: Jun proto-oncogene
KEGG: Kyoto Encyclopedia of Genes and Genomes
KIF11: kinesin family member 11
LCK: LCK proto-oncogene
MAD2L1: mitotic arrest deficient 2 like 1
MF: molecular function
PPI: protein–protein interaction
PTPRC: protein tyrosine phosphatase receptor type C
qRT-PCR: quantitative real-time polymerase chain reaction
RA: rheumatoid arthritis
STAT1: signal transducer and activator of transcription 1
VEGFA: vascular endothelial growth factor A
